# From beauty to knowledge: a new frame for the neuropsychological approach to aesthetics

**DOI:** 10.3389/fnhum.2015.00290

**Published:** 2015-05-20

**Authors:** Gianluca Consoli

**Affiliations:** Department of Philosophy and Social Sciences, University of RomeRome, Italy

**Keywords:** beauty, insight, expertise, knowledge, dis/fluency

Over the last decade the psychological and neurobiological approach to aesthetics has collected relevant data about the experience of art, aesthetic products, natural phenomena, and non-artistic objects—even if these data are somewhat divergent depending on the many differences in the stimuli, procedures, techniques, instructions, and tasks that are used (Chatterjee, 2011; Nadal and Skov, 2013). Given the strong historical association of the concept of beauty with art and aesthetics, the first applications of neuroimaging to visual aesthetic experience involved a privileged position for perceptual beauty (Cela-Conde et al., 2004; Kawabata and Zeki, 2004; Vartanian and Goel, 2004) but see also (Jacobsen, 2010; Ishizu and Zeki, 2013; Chatterjee, 2014). However, art history clearly shows that more often than not great artworks, especially modern ones, inhibit ordinary perceptual routines, violate predictions, involve disorder, disorganization, disharmony, ambiguity, contradictions, indeterminacy, uncertainty, strangeness, and so on (Bullot and Reber, 2013). Moreover, since Duchamp's use of everyday objects, the borders between art and non-art have been somewhat blurred, so that modern art requires a larger need for interpretation than any previous art (Leder, 2013). Finally, a given aesthetic object often serves a multiplicity of purposes for different people with different skills, in different contexts, and at different times (Nadal and Pearce, 2011).

In line with these features, the general focus of experimental studies has been rapidly and deeply reoriented. In particular, the neuropsychological approach to aesthetics has quickly gone beyond perceptual beauty and simple preference (Chatterjee and Vartanian, 2014). I propose that, if we consider with attention the more recent general trends of studies, at present the cognitive psychology and neuroscience of aesthetics are centered on aesthetic experience conceived as an experience of knowledge. First and foremost, this means that differences in processing experience influence aesthetic perception and evaluation—for instance, see the various studies concerning the effect of fluency on aesthetic appreciation (Reber et al., 2004). From this point of view, aesthetic experience is a function of previous knowledge and already acquired skills. However, recent evidence also shows that aesthetic experience represents at the same time a means of improving knowledge and enabling further skills acquisition. In this way, aesthetic experience is also cause and source of knowledge and skills. According to my point of view, this new perspective is undoubtedly shown by current behavioral, neuropsychological, and brain imaging data concerning three relevant and interconnected lines of inquiry: (a) gestalt formation and dis/fluent appreciation; (b) fiction and high-quality art; (c) experts/non-experts processing differences.

- Recent experiments concerning the aesthetic appreciation empirically demonstrate the deep relationship between perceptual insights and aesthetic pleasure. In the first study (Muth et al., 2012), photographs of cubist artworks by Picasso, Braque, and Gris were shown to participants without expertise in cubist art. The study was structured in two blocks, each showing the stimuli in a randomized order. During the first block, subjects had to rate the pictures on liking. During the second block, subjects rated how well they could detect objects within the artwork. All ratings were chosen from a 7-point-Likert-scale from 1 (“not at all”) to 7 (“very”). Data across participant revealed a strong relationship between the detectability of objects and liking, confirming that also in aesthetic perception form recognition is closely related to appreciation. In the second study (Muth and Carbon, 2013), two-tone images either containing a hidden form (i.e., a face) or not were repeatedly presented for half a second to participants. Stimuli were shown in a randomized order block-wise 13 times. The tasks alternated block-wise between choosing from a 7-point scale from 1 (“not at all”) to 7 (“very good”) how much one liked the picture and a detecting block. The latter comprised two ratings on a 1 plus 7-point scale (0: “no face recognized”; 7: “very clear”). Insight was defined by the highest gain in clearness between two subsequent blocks per participant and stimulus. All liking ratings per participant and block were then shifted in regard to their temporal occurrence relative to the insight block. Data clearly demonstrated that liking only significantly increased after having an insight; the intensity of insight, defined as degrees of clearness ratings, showed direct influences on the degrees of liking.
  This evidence supports the dis/fluent and dynamic conception of aesthetic appreciation. It is undoubtedly true that in general variables able to influence processing fluency (such as perceptual and semantic priming, stimulus repetition, and prototypicality) increase aesthetic appreciation (Forster et al., 2013). However, aesthetic appreciation often involves the success in establishing a new predictable pattern on a different level and so the transition from an initial state of uncertainty, associated with an unpleasant and negative affect, to a subsequent state of increased predictability, associated with an affective reward (Van de Cruys and Wagerman, 2011).
- Recent experimental results concerning fictional narratives indicates that the distinction between high-quality art and low-quality art can be empirically supported. Five experiments (with non-experts) show that reading literary fiction (such as De Lillo) temporarily enhances theory of mind, leading to a better performance in several well-established tests compared with reading popular fiction (such as the best-sellers of Gillian Flynn)—an activity that gives similar results to reading non-fiction and reading nothing (Kidd and Castano, 2013). For the first time scientific evidence suggests that (I) reading fiction really does have cognitive benefits, improving our social abilities; (II) aesthetic quality is determined by the different ways in which art involves readers, by guiding and prescribing different processing dynamics. Low-quality fiction typically tends to adopt ordinary templates, characterized by stereotypical and easily predictable patterns, with the goal of triggering intense emotions. The relevant information is familiar, it comes quickly and accurately to mind, it allows a fast recognition. Aesthetic appreciation is static: liking is a function of the fluency and ease of the interpreters' processing dynamics. In contrast, high-quality fiction typically tends to change conventional schemata, frustrating interpreters' expectations, with the goal of stimulating creative thought and disrupting stereotypes, biases, and prejudices. The relevant information is open to more than one interpretation, it enables a network of new and surprising associations and meanings, and it recursively prompts multiple cycles of perception and conceptualization. Aesthetic appreciation is dynamic: it is determined by an optimal amount of (un)predictability, which allows resolution to a recognized configuration (Van de Cruys and Wagerman, 2011; Bullot and Reber, 2013).
- Even when experts and non-experts appreciate the same artworks and share the same canon (Locher, 2011; Meskin et al., 2013), nonetheless their processing dynamics remain very different. Non-experts base their understanding on what is depicted (the content and their emotional reactions to it), whilst experts use art-specific classifications, related to prototypes of single artists or art schools (Augustin and Leder, 2006). Expertise increases liking and comprehension: compared to non-experts, experts like classical, abstract, and modern artworks more and find artworks more understandable (Leder et al., 2012). Finally, compared to non-experts, experts show attenuated emotional responses and like artworks more even when they perceive and judge negative and disturbing images: their emotional reaction is attenuated by attention to style and artistic execution (Leder et al., 2014). These findings show that the neural processes involved in aesthetic experience reflect interpreters' developmental and educational histories (Vartanian and Kaufman, 2013). In addition, they indicate that expertise represents the key component to understanding and appreciating dis/fluency.

In my reading, even if these lines of inquiry lack an overall integration, taken together they clearly suggest that the neuropsychological approach is increasingly framing aesthetic experience as a complex and multifaceted experience of knowledge—more precisely, as a specific implementation of the epistemic goal of knowing that becomes active when people experience objects (and not only artworks) adopting an aesthetic viewing orientation (Cupchik et al., 2009). This progressive shifting from the initial and privileged focus on perceptual beauty to the current relevance of concepts such as insight, gestalt formation, increased performance, meaning, understanding, and interpretation makes very difficult to reduce the scope of the neuropsychological approach to the restrictive study of the neural bases of beauty perception in art (Di Dio and Gallese, 2009).

## Conflict of interest statement

The author declares that the research was conducted in the absence of any commercial or financial relationships that could be construed as a potential conflict of interest.
